# Qualitative study on mental health screening needs of pregnant women in primary health care

**DOI:** 10.1016/j.mex.2024.103117

**Published:** 2024-12-20

**Authors:** Yuli Kusumawati, Izzatul Arifah, Eny Purwandari, Sukirman, Anindya Rintha Affindha

**Affiliations:** a Public Health Study Program, Faculty of Health Sciences, Univeritas Muhammadiyah Surakarta, Indonesia; b Master of Psychology Study Program, Faculty of Psychology, Univeritas Muhammadiyah Surakarta, Indonesia; c Informatics Engineering Education Study Program, Faculty of Teacher Training and Education, Univeritas Muhammadiyah Surakarta, Indonesia

**Keywords:** Needs, Screening of mental health, Pregnant women, Primary care, Qualitative Study on Mental Health Screening Needs of Pregnant Women in Primary Health Care

## Abstract

Mental health is currently a global problem in all age groups. the prevalence of mental disorders have not been described, especially pregnant women. This study aims to explore information about the need for screening and mental services provided in health centers and their recordings. This qualitative research was conducted with a phenomenological approach. This research was conducted at Gajahan, Kratonan, Penumping, Gilingan, and Sangkrah Health Centers. The main informants were midwives. Triangulation informants were 15 pregnant women, head of the health center and mental health workers. Data were collected through focus group discussions (FGD) and in-depth interviews. Data were analyzed using thematic analysis supported by Atlantis software. This study found three main themes, namely:

• The need for mental health screening for pregnant women, the implementation of mental health screening for pregnant women and related to reporting of mental health screening for pregnant women.
• Mental health screening for pregnant women in health centers is a very important activity and must be a priority to detect and prevent mental disorders in pregnant women.
• A policy is needed as a basis for implementing mental health screening for pregnant women with special recording and reporting.

## Graphical abstract

**Figure:**
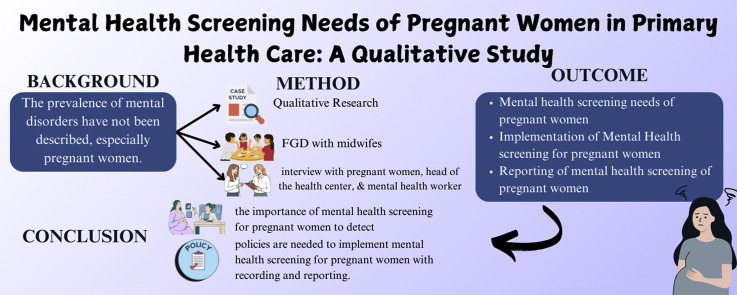

Specifications table

**Table:** 

Subject area:	Psychology
More specific subject area:	Mental health in pregnant women
Name of your protocol:	Qualitative Study on Mental Health Screening Needs of Pregnant Women in Primary Health Care
Reagents/tools:	Not applicable Triangulation of informants revealed that the youngest pregnant woman interviewed was 17 years old, while the oldest was 39. The maximum gestational age recorded was 37 weeks at the Kratonan PHC. Most women had attended antenatal care (ANC) 5–6 times. However, during ANC visits, none were asked to complete a questionnaire or discuss their mental health status.
Experimental design:	This qualitative research was conducted with a phenomenological cultural approach. The subjects of this study were midwives who provided ANC services at the health center as the main informants. The head of the health center and the manager of the mental health program became triangulation informants. Data were collected through focus group discussions (FGD) and in-depth interviews. Data were analyzed using thematic analysis supported by Atlantis software
Trial registration:	Not applicable
Ethics:	This study had obtained ethics approval from the Ethics Commission of the Faculty of Health Sciences, Universitas Muhammadiyah Surakarta No. 444/KEPK-FIK/VII/2024. Written informed consent was obtained from all potential participants before enrolment.
Value of the Protocol:	• Mental health during pregnancy is crucial to mitigate negative effects of mental illness. • Current data on mental health among pregnant women remains scarce. • Routine data collection is essential to tailor appropriate mental health services based on individual conditions and needs.

## Background

Mental health problems often occur and increase every year such as anxiety, stress, and depression which are influenced by multifactors. Approximately 10% of pregnant women and 13% of postpartum women worldwide experience mental disorders, particularly depression. In developing countries, these rates rise to 15.6% during pregnancy and 19.8% after childbirth [1]. In Indonesia, about 15.6 million women are affected. Mental health issues during pregnancy pose significant challenges in low- and middle-income countries as Sri Lanka, Afghanistan, Uganda, Kamboja, Nepal and Indonesiadue to inadequate screening and management strategies [2].

Indonesia lacks regular mental health data, relying mainly on infrequent five-year studies. Addressing mental health effectively requires both active and passive monitoring [3]. In Central Java, emotional disorders have a prevalence of 7.71%, with women (9.3%) affected more than men (6.9%). In Surakarta City, the prevalence is 5.51% [4,5]. Among individuals aged 15 and older in Central Java, depression rates are 5.11% for women and 3.66% for men. Mental health issues persist across the population, but data on high-risk groups like pregnant women is limited. A preliminary study in Surakarta found that 25.3% of pregnant women experienced symptoms of pregnancy-related depression [6].

Lack of a partner or social support, history of abuse or domestic violence, personal history of mental illness, unintended or unwanted pregnancy, negative life events and high perceived stress, current or previous pregnancy complications, and pregnancy loss were the most pertinent factors linked to antenatal depression or anxiety [[7], [8], [9]]. Mental health issues like anxiety and depression often worsen during pregnancy, negatively impacting both women's health and fetal development. These conditions can hinder women's ability to meet their physical needs and care for their babies. Depression during pregnancy can lead to severe sadness and anxiety, making it difficult for women to bond with or breastfeed their infants [10]. Symptoms of depression during pregnancy are sometimes not known for sure because during ANC, midwives only conduct anamnesis by interviewing, but cannot yet know for sure whether pregnant women are experiencing mental health problems.

Preliminary studies indicate that data on mental health progress in primary health services is lacking. Additionally, Maternal and Children's Health (MCH) services at primary health centers do not specifically address mental health for pregnant women, as their conditions remain uncertain. Routine data collection is essential for providing appropriate mental health services to pregnant women based on their specific needs. This study aims to explore the mental health services available at health centers in Surakarta City, assess the need and barriers for mental health screening for pregnant women.

### Description of protocol

This qualitative research was conducted with a phenomenological cultural approach. The main informants of this study were midwives who provided ANC services at the health center. The head of the health center and the manager of the mental health program became triangulation informants. In this study, midwives were the main informants because they serve pregnant women directly during ANC, so they are expected to know the initial conditions and symptoms that appear. Although not mental health professionals, midwives are responsible for ANC and are the first point of contact for pregnant women at the primary health care. In Surakarta, where health centres do not have psychologists, midwives act as early detectors of symptoms of emotional mental disorders. They often encounter problems of anxiety and depression in pregnant women, so support through routine screening is needed.

### Participant

#### Characteristics of research location

Data were collected from five community health centers in Surakarta City, Central Java, Indonesia. The selection of community health center locations was based on the criteria of representative community health centers for inpatient care, community health centers that had been used for previous research on Pregnant Women's Health, and community health centers that had never been the location of similar research. The community health centers selected as locations were Sangkrah, Gajahan, Kratonan, Penumping, and Gilingan Community Health Centers.

### Samples and recruitment

The inclusion criteria of the participants in this study were midwives who were assigned to provide non-inpatient ANC services or midwives who provided delivery services at inpatient health centers. Eligible participants were recruited using purposive sampling using the snowballing approach to achieve maximum variation in age, length of service, and education. A total of 38 eligible midwives were included in the data collection process using the Focus Group Discussion (FGD) method. The FGD was held in each health center for about an hour. The study conducted method triangulation with in-depth interview techniques with 15 pregnant woman. Data collection was carried out during July and August 2024.

### Research instruments

The FGD and interview guides were developed based on literature regarding Mental Health guidelines, General Mental Health Screening, and Pregnant and Postpartum Women [11] (Beck et al., 2022). Four experts—psychologists, mental health nurses, midwives, and a health center head—participated in the FGD, which focused on the implementation of the Mental Health program, screening processes, and the handling, recording, and reporting of mental health issues in pregnant women. Researchers also explored the need for mental health screening in this population. The interview questionnaire was created based on the Indonesian Antenatal Care (ANC) service standard (K10), which mandates the integration of mental health screening into ANC services. However, researchers found no information on the mental health status of pregnant women at the PHC. Thus, they sought insights on the implementation of mental health screening, methods and instruments used, perceptions of routine screening's importance, and barriers encountered.

## Data collection

The FGD was conducted from July to August 2024 in each health center, guided by the main researcher, in Indonesian and assisted by research members, research assistants (Indonesian as the female language) and assisted by facilitators. Each FGD lasted approximately 60 min. The FGD for midwives was conducted in five groups. After five FGDs, no new information was found, so data collection was stopped. Researchers and facilitators anonymously tracked and transcribed the Focus Group Discussion (FGD) process. Observers recorded non-verbal elements of the FGD in a logbook.

Furthermore, data collection was carried out by conducting in-depth interviews individually with three heads of primary health centers (PHC) and three mental health program officers, each via Zoom meeting, each interview was approximately 45 min. All participants signed an informed consent after receiving an explanation of the purpose of the study before the interview began. In-depth interviews to explore personal experiences in managing mental health programs at health centers. When no new information was found, data collection was stopped, and the researcher concluded that saturation had occurred. Saturation was achieved after the study collected information from 38 midwives through FGDs, three heads of health centers, three mental health workers, and 16 pregnant women through in-depth interviews. For further data analysis, all audio recordings from the Focus Group Discussion (FGD) and in-depth interviews were transcribed verbatim for further data analysis. For further data analysis, all audio recordings from the Focus Group Discussion (FGD) and in-depth interviews were transcribed verbatim for further data analysis.

### Data processing

The qualitative data analysis process was discussed by the research team. Audio recordings and interviews from the Focus Group Discussion (FGD) were transcribed verbatim. In addition, relevant groupings and codes were coded. The results were grouped, abstracted into specific categories, and then analyzed using thematic analysis. The themes that emerged from the Focus Group Discussion (FGD) and in-depth interviews have been described in selected excerpts to be translated into English. The organization of codes into relevant categories and themes was done using Atlantis software.

To ensure the validity of the data analysis, the authors first analyzed the content of each transcription into codes, categories, and themes. Next, the authors monitored the transcriptions, codes, categories, and mergers, and then discussed and agreed on the results. Finally, the authors analyzed all the data that had been entered into categories and themes. All authors conducted peer debriefing, understood the analysis method, and agreed with the results of this study.

Four principles of increasing trust in qualitative research results, namely credibility, transferability, dependability, and certainty (confirmability) have been used by the author. Increasing the credibility of this study has been done by checking interpretations with participants. In addition, researchers checked information based on the results of Focus Group Discussions (FGD) and previous interviews to ensure the credibility of the data collection process. The author considered the ideas that had been changed with the next participant. To increase dependability and confirmability, the authors from the research team assessed each other. The authors discussed to determine codes and categories and reached a consensus on the themes found.

### Ethical considerations

The researcher received ethical approval from the Ethics Commission of the Faculty of Health Sciences, Universitas Muhammadiyah Surakarta No. 444/KEPK-FIK/VII/2024 before the data was collected. Permission to conduct data collection was obtained from the Surakarta City Research and Development Agency, the government agency responsible for the research, the Head of the Surakarta City Health Office, and the place where the research was conducted.

### Protocol validation

This study was conducted in five health centers in the Surakarta City area. The FGD was conducted in each health center. The largest number of FGD participants came from the Gajahan health center, namely 10 people, consisting of five midwives in inpatient care and five outpatient midwives in ANC services. The mean age of midwives across the five FGD groups was similar, with the Penumping Health Center group having the highest average at 39.71 years. The Gilingan Health Center group reported the longest average service length of 15.17 years, with a maximum of 34 years. Most midwives held a Diploma 3, while there was one midwife each with a Bachelor's and Professional education. Detailed data is shown in Table 1.

**Table 1:** Data on Characteristics of FGD Participant Informants.

Health Center Location	Number of Informants (people)	Mean of Age Min-Max (year)	Mean of Working Period Min – Max (Year)	Education
Gajahan	10	38.6 26 - 49	14.1 2 months – 30 years	1 Bachelor's degree 1 Profession 8 Diploma 3
Sangkrah	7	36.43 29 - 55	9.17 1 – 30	7 Diploma 3
Kratonan	6	36.67 29 - 48	11.2 2 months – 22 years	1 Bachelor's degree 1 Diploma 4 4 Diploma 3
Penumping	7	39.71 29 – 56	13.6 2 months – 37 years	7 Diploma 3
Gilingan	8	39.14 31 - 55	15.17 2 months – 34 years	1 Diploma 4 7 Diploma 3

Midwives' work experience in ANC services influenced their responses during the interview on mental health screening of pregnant women. Experienced midwives tended to give detailed responses and share their experience of referring pregnant women with mental disorders. Younger midwives, despite not having much experience in ANC and pregnant women's mental health cases, responded positively to the need for online screening, which was perceived to facilitate the detection of mental problems. Meanwhile, older midwives prefer using paper to speed up action.

Interviews with midwives aimed to evaluate mental health screening of pregnant women in ANC services. The author also interviewed mental health workers to triangulate information. The results show that mental health screening has not been fully implemented, only on pregnant women who show early symptoms based on behavior and responses during ANC on referral from midwives (Table 2).

**Table 2:** Data on Characteristics of Informants of Heads of Health Centers and Mental Health Officers.

Initials	Age (yrs)	Length of Service (yrs)	Position
An	37	13	Head of PHC
Nr	49	18	
Frh	39	15	
Id	57	13	Mental Health Officer
AKS	34	9	
Dw	35	10	

From the characteristic data, it shows that there are 3 heads of health centers where the highest age is 49 years old with the longest work period of 18 years. While for mental health workers, the highest age is 57 years old with the longest work period of 13 years. An interview with the head of PHC showed that the hospital only accepts referrals for severe mental illness from the (PHC). PHC cannot identify pregnant women who need to be referred due to mental disorders without measurement. If there is only information from the family or cadres, there is no serious action to ascertain the patient's condition and provide intervention (Table 3).

**Table 3:** Characteristics of Informants in the Triangulation Study of Pregnant Women.

Initial	Age (yrs)	Gestational Age (weeks)	Location of PHC
RAO	22	37	Kratonan
MI	32	28	
TN	18	14	
NMA	39	32	Gajahan
WA	29	18	Sangkrah
Mry	34	12	
PM	29	12	
SH	35	30	Penumping
RAP	34	29	
VM	26	34	
DHS	17	35	Sangkrah
LNA	26	32	
AL	28	30	
Dy	35	13	Gilingan
Cnt	24	29	

Triangulation of informants revealed that the youngest pregnant woman interviewed was 17 years old, while the oldest was 39 years old. The highest of gestational age was 37 weeks at the Kratonan PHC. Most women had attended antenatal care (ANC) 5–6 times. However, during ANC visits, none were asked to complete a questionnaire or discuss their mental health status.

This study uncovered three main topics, namely the Need for Mental Health Screening of Pregnant Women, Implementation of Mental Health Screening of Pregnant Women, and Reporting of Mental Health Screening of Pregnant Women. Table 4 showed the complete results of the qualitative analysis.

**Table 4:** Results of qualitative data analysis.

Theme	Category	Coding
Mental health screening needs of pregnant women	Service needs	Mental health screening is essential for pregnant women.
		Specialized instruments are required to assess the mental health of pregnant women.
		Innovations are needed from health centers to enhance services.
		Support for stunting interventions is necessary.
		Improvements in women's and children's health are needed.
		Psychologists should be available at health centers.
		Mental health training for midwives is essential.
		There is a need for mental health policies and regulations specifically for pregnant women.
Implementation of Mental Health screening for pregnant women	Mental Health Screening for Pregnant Women	Not all health centers provide mental health screening for pregnant women.
		The Kratonan PHC has recently been implemented.
		Currently, there is no mental health screening in Penumping PHC
	Mental Health Problem Indicators	The midwife remains uncertain.
		Patient's narrative.
		Patient's facial expression.
		Observe patient gestures.
		Conceal psychological issues.
		Denial of pregnancy.
		Crying suddenly.
	Mental Health Instruments	No specialized instruments for pregnant women are available, and this is still unknown.
		Currently using the SRQ.
		Utilizing the SDQ.
	Screening Challenges	Need a special screening room
		Need additional human resources
		Limited inspection time
	Screening Executor	Screening implementer: midwife
		Screening implementer: nurse
Reporting of mental health screening of pregnant women	Special reporting	Mental health reporting has so far used the ‘simkeswa’ system
		Reporting only for diagnosis of severe mental disorders
		Selective reporting of screening results
		High reporting of screening results in poor areas
		Transparent reporting of screening results
		There is no specific template for pregnant women's mental health reporting

### Mental health screening needs of pregnant women

All participants agreed that mental health screening for pregnant women is important and needs to be included in the ANC service package. These needs include regulations, special instruments, training for midwives, and human resources for health center psychologists. Participants from health workers said that mental health screening for pregnant women can be an innovative program for health centers, including supporting stunting interventions and improving women's and children's health.

Research findings indicate that several health centers in Surakarta City have not implemented specialized mental health screening for pregnant women, with screenings currently focused on general patients. Midwives occasionally learn about pregnant women with mental health issues through community health cadres, as noted by several midwife informants during the FGD:

Just interview and maybe there is …. if we are from the cadres, we are usually given leaks (information). “that woman” we usually see oh this is it. The cadres are there “Ma'am, she doesn't have a husband”, “Ma'am, it's an unwanted pregnancy” The cadres already know that. … well, we often go to the integrated health post, and we can, often go to the cadres. If the others are forced to interview, we mean we see what the problem is (Midwife 1, 49 years old). There is no checklist yet, there is no initial screening for mental health… (Midwife 2, 42 years old).

Additionally, the midwife's information is supported by the pregnant women's statement that she has never received mental health screening, as illustrated in her comments below:

…not yet ma'am, I have never filled out a questionnaire like that… (pregnant woman, 39 years old).

…not yet ma'am… (pregnant woman, 26 years old).

Mental health screening has been done generally on patients as needed and on adolescents, while on pregnant women it has never been done specifically. This is in accordance with the information provided by the midwife as follows:

…At the health center, there are no services specifically for mental health yet, ma'am… (Midwife, 40 years old).

…Nowadays, the health center is a life cycle, yes, this is my plan from ANC, from the first time the woman comes, the first time she comes and the five-month-old who comes, we will give health screening (SRQ)-20. That is my plan, ma'am… (Mental Nurse, 34 years old).

This information is also reinforced by information conveyed by the head of the health center, as in the following statement:

…So every year we have a target to be able to conduct mental health screening. What we use is the screening form that we have received from the Ministry of Health, namely SRQ, and SDQ, …. and the target is more for the general public, so there is nothing specific that we have done for pregnant women… (Head of PHC, 37 years old).

…for pregnant women, mental health services have not been carried out specifically, there have been none so far, there is no routine screening for pregnant women for mental health, …… has not been done…. (Head of PHC, 39 years old).

The study results obtained information that screening of the mental health of pregnant women needs to be done. Because by knowing the mental condition of pregnant women, then in the ANC examination, midwives can provide counseling services themselves or need to be referred. This is as seen in the statement made by the following midwife:

…Needed, because it will affect later, … during pregnancy care, the delivery process, and later during baby care, it is very necessary. If for example it had been detected earlier, … maybe there could have been assistance or referral to a higher level… (Midwife 3, 39 years old).

## Implementation of mental health screening

### Mental health screening for pregnant women

The study results showed that mental health screening that has been carried out in health centers is general mental health screening, not specifically measuring mental health problems of pregnant women. Kratonan PHC and Gilingan PHC are 2 health centers that have just tried to implement mental health screening for pregnant women. In general, using a questionnaire that has been trained on mental health program holders. While screening for pregnant women has not been carried out, because it is not one package with the MCH section. This is as conveyed by the midwife as follows:

….Not yet, because MCH we follow the standard from the Ministry of Health which is 10T, ma'am. If the 10T is only included in counseling according to needs, ma'am, well that's what we do…. (Midwife4, 34 years old).

### Mental health problem indicators

Nearly all midwives have encountered pregnant women with mental health issues, but they often remain uncertain. They suspect problems based on the patient's facial expressions, sad gestures, and nonsensical responses. If a pregnant woman shows such signs during ANC, the midwife conducts an in-depth interview. Patients sometimes conceal their psychological struggles, crying unexpectedly and only sharing their feelings when they struggle with their pregnancy. This aligns with the following midwife's comments:

….Yes, physically, she usually suddenly cries. Then, what is it called, she is not open…..when we do the anamnesis, she already has teary eyes, then finds it hard to express herself, oh from there sometimes we can judge that this means there is a problem with this pregnancy. Usually, we dig…..she doesn't want to get pregnant anymore. So there she finds it hard to accept the pregnancy, there we try to manage her emotions….. (Midwife5, 36 years old).

### Mental health instruments

In several health centers that have conducted mental health screening, the instruments used for screening so far are still common, namely SDQ for adolescents and SRQ for adults. Mental Health Screening is included in the essential Non-Communicable Disease (NCD) program. Midwives said they did not know that there was a special Mental Health instrument for pregnant women, so far they have been using the SDQ and SRQ instruments according to instructions from the Ministry of Health and have been trained for the holders of the Mental Health program at health centers. So midwives also use that instrument. This is in accordance with the results of the midwife's FGD which were conveyed in the following statement:

…… is it true that in midwifery services it has been given to pregnant women? If with a questionnaire not yet….. Not yet, only interviews…. (Midwife5, 35 years old).

……For Gilingan Community Health Center, there is screening, but for special mental health services, there is not yet…. There is already, ma'am, yes, it's the same, because all health centers are the same, there is already SRQ 21 screening, and there is also pregnant women but it is still limited to pregnancy classes (midwife6, 30 years old).

….just recently we had a screening…. given a questionnaire to pregnant women to detect mental health, we use SRQ…. (Head of PHC 2, 49 years old).

### Screening challenges

Although several PHC have conducted general mental health screening, several informants stated that mental health screening has not been implemented because there are several obstacles or challenges, including the need for a special room and the need for additional personnel because examination time is limited. This is also reinforced by triangulation informants of pregnant women who did ANC.

…..No, I have never been asked to fill out questions about feelings of happiness with pregnancy, questions about…. ever crying alone….like that, never…. (Pregnant Woman 2, 29 years old).

### Screening implementation

Mental health screening is conducted by program officers for general patients inside the building and for teenagers outside, such as in schools. If screening for pregnant women is implemented, midwives or nurses can conduct it in coordination with mental health officers. This aligns with statements from mental health officers during interviews (Table 5).

…….To be specific, mental health services themselves do not exist yet, we from the Penumping Community Health Center, right….we have a program like me as the mental health program holder, so later if there is a patient who needs mental screening, I will immediately consult a doctor (Mental Health Officer3, 34 years old).

**Table 5:** Observation result.

Informan	Challenges/barriers to mental health assessment	Possible solution
Midwives	1. General mental health screening using the SRQ paper. 2. Filling in while waiting in the queue 3. Not all patients at the health centre have a mobile phone 4. Special paper instruments for pregnant women must be counted manually, but interventions can be given immediately. 5. Excessive duties of midwives,	1. Screening using simple instruments specific to pregnant women 2. The questionnaire can be paper-based, 3. Questionnaire in the form of an application 4. Health centre can provide phone cell. 5. The application can add educational features in addition to direct measurement.
Mental Health Officer	1. Mental health screening, still general, general patients and adolescents, while pregnant women have not been routinised, only those suspected 2. Reporting in general, through the Ministry of Health's system, there is no specialised reporting for pregnant and postpartum women.	1. Integration with Antenatal Care services is required 2. All pregnant women are asked to complete the Mental Health Questionnaire during ANC, at least at the first visit (K1) and before delivery (K6).
Head of primary health care	There is no policy to mandate mental health reports specifically for pregnant women.	1. A policy from the Health Office is required. 2. A special mental health report can be developed for pregnant and postpartum women.

### Mental health screening reporting for pregnant women

#### Special recording

This study found that specific mental health screening for pregnant women is lacking, with only general screenings in place. There is no dedicated recording or reporting template for mental health issues in pregnant women. If a pregnant woman is identified with a mental health disorder, whether through anamnesis or the SRQ questionnaire, it is recorded as a single case by the mental health program holder, as confirmed by the midwife's statement:

…during the anamnesis, there was also the potential to lead to mental disorders due to the condition of the pregnancy, but indeed because in our regulations, in our SOP, there is no… what is special recording, so we do not have any recording for the mental health of pregnant women… (midwife, 38 years old).

So far, recording and reporting of mental health problems carried out by mental health program officers is only for severe mental disorders. In addition, no policy requires screening and reporting of mental health specifically for pregnant women. This is in accordance with what was conveyed by the triangulation informant for mental health officers as follows:

…I also reported to SIMKESWA (mental health system information), the ones to the Ministry of Health are only ODGJ (Schizophrenia), shackles, and ODMK also exist, later on, those who are not in the top five can also be reported, it's just that the service only focuses on those with severe ODGJ, shackles, suicide, and those who are on drugs…. (MH Officer, 34 years old).

Mental health screening for pregnant women also does not have a special template, and because there is no obligation, it is not reported, only in the MCH book records, it is marked as high risk. This is in accordance with the information provided by the midwife, as follows:

Not reporting, ….

Because it was not requested, so we did not report it. Internal handling only but for general mental health reports, the Health Service…. requested it. But if it was… for example, the one that was included in mental health, the reporting was in collaboration with mental health. the report was included in mental health, if the report was in our MCH… the high-risk pregnant women were included… (Midwife, 48 years old).

This finding aligns with interviews with mental health officer, indicating that there is currently no reporting system or specific template for mental health issues among pregnant women, as detailed below:

….if reporting records already exist, women. only if there isn't one specifically for pregnant women. So like in…. eh, that's the whole thing, like, for example, schizophrenia, then patients who are depressed, anxious, so that comes in there.

…… it is not specific to depressive disorders in pregnant women, there are none yet. So later if there are patients with depression or uh anxious patients, they will be included in the general category (MH Officer, 34 years old).”.

## Discussion

The mental health of pregnant women is currently very necessary, considering that pregnant women are a vulnerable group to experience mental health problems, such as anxiety and depression. Although depression is a common medical condition in women during the perinatal period, it is related and has serious impacts. Untreated perinatal depression in women can cause low birth weight and impaired social, cognitive, and emotional development in babies [10]. Given the major impact of mental health problems on women and their fetuses, mental health screening during pregnancy is very important as part of primary health care.

In Indonesian cultural norms, women are expected to obey their husbands and parents, including during pregnancy. However, some conditions during pregnancy are considered common or taboo by parents [[9], [10], [11]]. Previous research shows that emotional disturbances in pregnant women are considered normal, and unstable emotions are often ignored by the environment [15]. Midwives also observed that pregnant women often receive myths of prohibition, such as not eating fish [[12], [13], [14]]. Women pregnant have Experienced domestic violence. The anxiety experienced by pregnant women was also often caused by a less supportive family environment. Women pregnant have a traumatic experience obstetric [15]. This research finding not all pregnant women undergo mental health screening assessments; only those with early symptoms are assessed. As a result, current ANC services do not recognize and record the overall health condition of pregnant women. Current protocols only include general mental health screening for patients and adolescents in schools. Measurement of mental disorders in pregnant women is done only if there are early symptoms, with no specific reporting obligations. Routine screening for all pregnant women in ANC has not been implemented.

Screening is essential to determine if a pregnant woman exhibits symptoms of psychological disorders. According to Niel and Payne, screening is crucial for diagnosing perinatal depression. Therefore, mental health screening should be conducted at least during the first ANC visit [10]. Several informants noted that screening is performed at least once at the beginning of ANC, with the possibility of repeating it at subsequent visits before and after delivery. If a pregnant woman's screening score exceeds the standard, counseling or referral can be provided. Midwives should be aware that postnatal depression (PND) can continue after ANS. ANS screening has not received the attention that PND has although the effects may be similar [16].

In this study, several health centers that have implemented mental health screening are still general in adult and adolescent patients. The screening instruments used are the Strengths and Difficulties Questionnaire (SDQ) and the Self-Reporting Questionnaire (SRQ). The SDQ is a questionnaire to evaluate behavior with a focus on strengths and difficulties specifically for children and adolescents aged 3 to 17 years [17]. The SQR was developed by the World Health Organization (WHO) for psychiatric disorder screening and research purposes. Midwives and mental health program officers are not yet aware of any mental health questionnaire specifically for pregnant women. Midwives hope that mental health screening for pregnant women can use a shorter questionnaire.

Interview results show that mental health screening still uses paper-based instruments, which require manual calculations to obtain results. According to the senior midwife, paper instruments allow for quick results and immediate intervention. However, manual measurements and actions given need to be recapitulated, causing reports to be delayed. Although there are screening instruments in the form of Google Forms, barriers appear as some pregnant women do not have mobile phones or are reluctant to disclose their problems.

Many previous studies have measured the mental health of pregnant women, with the Edinburgh Postpartum Depression Symptoms (EPDS) questionnaire [18]. In previous studies, the EPDS questionnaire has been widely used to screen for depression in general women, women during pregnancy, and the first year after giving birth [17]. The EPDS questionnaire is simpler, consisting of 10 questions with graded answer choices. Midwives suggest that if there is a simpler and shorter questionnaire, it can be used for screening so that the time needed to fill it out is faster. In the future, it can be recommended to use the EPDS questionnaire for mental health screening of pregnant women in health centers. The screening results obtained are more specific to pregnant women, after the score is known > 13, counseling intervention can be immediately given by a trained midwife, or psychologist or referred to a specialist. One of the popular tools for identifying postnatal anxiety and depression is the Edinburgh Postnatal Depression Scale (EPDS). EPDS is a reliable screening instrument of choice because of its validity and recommendations in clinical guidelines [19,20].

The previous study noted that 25.3% of pregnant women in Surakarta City experienced antenatal depression [6]. Pregnant women in interviews stated that they had never been asked or given information related to mental health. The results of mental health screening that have been carried out so far at the health center have been reported to the health office and the data is directly summarized on the website of the Ministry of Health. However, the reports are still global or general, only based on age groups. So the prevalence of mental health data for pregnant women has not been seen. Recording and reporting the results of mental health screening for pregnant women specifically aims to find out the actual incidence of pregnancy depression. Reporting of mental health screening is carried out by health center officers only for the diagnosis of severe mental disorders.

Mental health disorders such as depression and anxiety are common in pregnant women, but these disorders often cannot be categorized as severe, because they do not require treatment. This causes depression and anxiety to be not included in the reported mental health reports. In addition, the results of in-depth interviews stated that because there is no policy and obligation to report the results of screening for pregnant women, there is no special reporting for pregnant women. Midwives can know a pregnant woman's feelings during ANC. If the pregnant woman responds poorly, the midwife will report the evaluation to a nurse or psychiatrist for further assessment and intervention. However, not all pregnant women are open about their problems, and the number of midwives conducting ANC examinations is limited. Although midwives feel the need to screen for mental health, this is not done for all pregnant women as there is no mandatory reporting.

Bronfenbrenner's ecological systems theory involves five systems: microsystem, mesosystem, exosystem, macrosystem, and chronosystem. The development of mental health screening for pregnant women must consider all these systems. At the microsystem level, family and partner support is crucial; at the mesosystem level, interactions with social environments and healthcare services are important; at the exosystem level, economic, political, and cultural factors are significant; at the macrosystem level, social norms and community values are relevant; and at the chronosystem level, physical, emotional, and psychological changes during pregnancy need to be considered. Therefore, mental health services should be developed to meet the needs of individuals and their social environments [21].

These results are consistent with research findings in Vietnam on the lack of comprehensive mental health policies. The absence of comprehensive policies and guidelines on mental health in the health system is a barrier to including mental health services in routine antenatal visits [22]. Government policies or regulations still play an important role in mental health reporting. If there is no policy for mandatory reporting, then the screening results may not be reported. This makes it difficult to get a true picture of the mental health of pregnant women. It is hoped that reporting the results of this screening can be used to identify risk factors for mental health problems that occur. In addition, it can monitor the development of mental health problems in pregnant women if they have received intervention from health workers.

## Limitations

This study only explored midwives' perspectives on ANC services and the implementation of mental health screening of pregnant women, where midwives felt necessary but not burdened by the task. We only interviewed a few pregnant women for triangulation, and the results showed that they were never asked to fill out questionnaires on mental health measures. The results cannot be generalized; they only apply to the informant's location, where according to the perceptions of midwives, health workers, and the head of the primary health center, mental health screening has been conducted in general, but specifically for pregnant women has never been reported because there are no reporting instructions.

## Conclusion

Mental health screening of pregnant women should be routinely conducted in primary care integrated with ANC, so that those experiencing mental health problems can receive prompt intervention. Policies are also needed to ensure regular reporting of pregnant women's mental health. In the future, routine screening is needed, with simple and easy tools, as well as recording and reporting facilities to monitor it.

## CRediT authorship contribution statement

**Yuli Kusumawati:** Methodology, Investigation, Formal analysis, Writing – original draft. **Izzatul Arifah:** Conceptualization, Validation, Writing – review & editing. **Eny Purwandari:** Methodology, Writing – review & editing, Resources. **Sukirman:** Conceptualization, Investigation, Software, Writing – review & editing. **Anindya Rintha Affindha:** Investigation, Formal analysis, Writing – review & editing.

## Declaration of competing interest

The authors declare that they have no known competing financial interests or personal relationships that could have appeared to influence the work reported in this paper.

## Data Availability

The authors do not have permission to share data.

## References

[1] de Almeida C.S., Miccoli L.S., Andhini N.F., Aranha S., de Oliveira L.C., Artigo C.E. (2016). Comprehenship mental health action plan 2013-2023. Rev. Bras. Linguíst. Apl..

[2] Agampodi T., Amarasinghe G., Wickramasinghe A., Wickramasinghe N., Warnasekara J., Jayasinghe I. (2023). Incorporating early pregnancy mental health screening and management into routine maternal care: experience from the Rajarata Pregnancy Cohort (RaPCo), Sri Lanka. BMJ Glob. Health.

[3] Zhou W., Xiao S. (2015). Existing public health surveillance systems for mental health in China. Int. J. Ment. Health Syst..

[4] Kemenkes (2018).

[5] Kemenkes R.I. Profil Kesehatan Indonesia 2021. Pusdatin.Kemenkes.Go.Id. 2022. Kementrian Kesehatan Republik Indonesia.

[6] Kusumawati Y., Salsabila D.A., Widyawati W., Dewi F.S.T. (2022). Depression and anxiety as mental health disorders that affect pregnancy: a case study in Surakarta, Indonesia. J. Med. Chem. Sci..

[7] Biaggi A., Conroy S., Pawlby S., Pariante C.M. (2016). Identifying the women at risk of antenatal anxiety and depression: a systematic review. J. Affect. Disord..

[8] Dadi A.F., Akalu T.Y., Wolde H.F., Baraki A.G. (2022). Effect of perinatal depression on birth and infant health outcomes: a systematic review and meta-analysis of observational studies from Africa. Arch. Public Health.

[9] Mainali A., Infanti J.J., Thapa S.B., Jacobsen G.W., Larose T.L. (2023). Anxiety and depression in pregnant women who have experienced a previous perinatal loss: a case-cohort study from Scandinavia. BMC Pregnancy Childbirth.

[10] Van Niel M.S., Payne J.L. (2020). Perinatal depression: a review. Clevel. Clin. J. Med..

[11] Beck A., Hamel C., Thuku M., Esmaeilisaraji L., Bennett A., Shaver N. (2022). Screening for depression among the general adult population and in women during pregnancy or the first-year postpartum: two systematic reviews to inform a guideline of the Canadian Task Force on Preventive Health Care. Syst. Rev..

[12] Agus Y., Horiuchi S., Porter S.E. (2012). Rural Indonesia women's traditional beliefs about antenatal care. BMC Res. Notes.

[13] Zulkifli Z., Yenni, Sari D.Y., Rachel A., Sasihade D.E. (2023). Pregnancy tradition ceremony in Javanese society. Indones. J. Med. Anthropol..

[14] Rachmayanti R.D., Diana R., Anwar F., Khomsan A., Riyadi H., Christianti D.F. (2023). Culture, traditional beliefs and practices during pregnancy among the Madurese tribe in Indonesia. Br. J. Midwivery.

[15] Kusumawati Y., Widyawati W., Dewi F.S.T. (2022). Vulnerable to mental health problems: pregnant women and husband's perception in Surakarta, Indonesia. Enferm. Clin..

[16] Martin C.J.H., Norris G., Martin C.R. (2023). Midwives’ role in screening for antenatal depression and postnatal depression. Br. J. Midwivery.

[17] Black S., Pulford J., Christie G., Wheeler A. (2010). Differences in New Zealand secondary school students’ reported strengths and difficulties. NZ J. Psychol..

[18] Oliveira T.A., Luzetti G.G.C.M., Rosalém M.M.A., Mariani Neto C. (2022). Screening of perinatal depression using the Edinburgh postpartum depression scale. Rev. Bras. Ginecol. Obstet..

[19] Bayrampour H., McNeil D.A., Benzies K., Salmon C., Gelb K., Tough S. (2017). A qualitative inquiry on pregnant women's preferences for mental health screening. BMC Pregnancy Childbirth.

[20] Blackmore R., Boyle J.A., Gray K.M., Willey S., Highet N., Gibson-Helm M. (2022). Introducing and integrating perinatal mental health screening: development of an equity-informed evidence-based approach. Health Expect..

[21] Crawford M. (2020). Ecological systems theory: exploring the development of the theoretical framework as conceived by bronfenbrenner. J. Public Health Issues Pract..

[22] Ford E., Roomi H., Hugh H., van Marwijk H. (2019). Understanding barriers to women seeking and receiving help for perinatal mental health problems in UK general practice: development of a questionnaire. Prim. Health Care Res. Dev..

